# Increase in b-wave amplitude after light stimulation of the blind spot is positively correlated with the axial length of myopic individuals

**DOI:** 10.1038/s41598-022-08319-5

**Published:** 2022-03-21

**Authors:** Tim Schilling, Ana Amorim-de-Sousa, Nikita A Wong, Hamed Bahmani, José Manuel González-Méijome, Paulo Fernandes

**Affiliations:** 1 Dopavision GmbH, Berlin, Germany; 2 Clinical & Experimental Optometry Research Lab (CEORLab), Physics Center of Minho and Porto Universities (CF-UM-UP), University of Minho, Braga, Portugal; 3 Department of Physiology of Cognitive Processes, Max Planck Institute for Biological Cybernetics, Tübingen, Germany; 4 Bernstein Center for Computational Neuroscience, Tübingen, Germany

**Keywords:** Retina, Translational research

## Abstract

Altered retinal dopamine and ON-pathway activity may underlie myopia development. It has been shown that the stimulation of the blind spot with short-wavelength light increases the electroretinogram (ERG) b-wave amplitude of myopic eyes and may engage the retinal dopaminergic system. This study evaluated the impact of various durations of blind spot stimulation on the electrophysiological response of the myopic retina and their relationship to axial length. Six myopic individuals underwent three short-wavelength blue light blind spot stimulation protocols (10 s, 1 min, 10 min) using a virtual reality headset. As a control condition, no stimulation was shown for 1 min. The b-wave amplitude of the photopic full-field ERG was measured at baseline and 10, 20, 30, 40, 50, and 60 min after each condition. A significant increase in b-wave amplitude was observed for all stimulation protocols compared to the control. The peak b-wave amplitude was observed 20 min after the 1-min stimulation protocol and 60 min after the 10-min stimulation protocol. A significant positive correlation was found between axial length of the eye and percent change in b-wave amplitude for the 10-min stimulation protocol. A rapid and a delayed b-wave time course responses were observed following 1 min and 10 min of blind spot stimulation, respectively. Overall, these results indicate that light stimulation of the blind spot for various durations elevates ON-bipolar cell activity in the retina and as such is assumed to reduce the myopic response. These findings could have implications for future myopia treatment.

## Introduction

As the prevalence of myopia in children continues to increase^1^ there is growing interest in treatments to reduce the excessive axial elongation that is associated with myopia development and progression. Myopia due to excessive eye growth can have various adverse effects over the long-term^2^ and, as a result, slowing myopia progression has become a challenge for scientific and clinical communities around the world^3–5^. It is now widely assumed that the mechanism responsible for regulating eye growth and myopia progression is localized within the eye^6,7^. Yet which visual^8–13^ and non-visual^14–16^ factors are key to maintaining normal ocular growth remains up for debate.

In humans, the natural course of ocular growth in myopic eyes can be significantly modulated by way of optical devices in spectacles, contact lenses, and administration of atropine, as has been shown in recent randomized clinical trials^17–20^ and meta-analyses^21,22^. A lower incidence of myopia in children has also been associated with more outdoor activity^23,24^. One proposed mechanism for this association is increased retinal dopamine as a result of bright light exposure^25^. There is now a growing body of evidence in favour of an important role of the retinal dopaminergic system in ocular growth regulation and myopia development^14,26^.

Dysfunction of the retinal dopaminergic system might also be related to the proposed relationship between ON-pathway activity and myopia. Animal models lacking a functional ON-pathway have reduced retinal dopamine levels and are more susceptible to experimental myopia^27,28^. Previous results suggest that retinal ON- and OFF-pathways are also involved in refractive error development in humans^29,30^. Given that ON-bipolar cells are the primary input to dopaminergic amacrine cells (DACs), the main source of dopamine in the retina^31^, it is likely that changes in retinal dopamine and ON-pathway activity interact in the myopic eye^27,28^.

As the recipient of all incoming visual information and the central processing structure of the eye, the retina is likely responsible for the signalling necessary for eye growth regulation. If irregular DAC and ON-bipolar cell activity contribute to myopia development, these changes should be reflected in retinal activity. Retinal electrical activity can be assessed non-invasively using the electroretinogram (ERG). The b-wave of the light-adapted ERG waveform, in particular, reliably reflects the activity of ON-bipolar cells^32^ and can detect changes in ON-pathway functionality^33,34^. Thus, the ERG may provide a means through which to explore the potential retinal mechanisms of myopia development and control^35,36^.

Indeed, ERG studies have consistently reported a reduction in the b-wave amplitude of individuals with myopia, compared to emmetropic individuals^37–39^. Further research has determined that the amplitude of the b-wave is inversely associated with axial length^39,40^. Studies have also shown that the b-wave implicit time is prolonged in individuals with high myopia^41^. Other studies have found that the b-wave amplitude for the rod pathway increases with refractive error^36^, but is significantly decreased for all three cone pathways in myopia^42^. Moreover, the central induced component of the multifocal ERG, which primarily reflects activity in the inner retina, is significantly correlated with refractive error changes in children, such that a reduction in inner retinal electrical activity appears to precede myopia development in childhood^43^. On the other hand, dual focus contact lenses, which have been used to slow the rate of myopia progression in children, increase the amplitude of the induced component^44^. Overall, it appears that there is some functional change in the myopic retina that is likely localized in the inner retina and that may be related to changes in the retinal dopaminergic system^35,36^.

Recently, we have reported significant changes in the ERG and pattern ERG (PERG) responses following stimulation of the blind spot with short-wavelength light^45^. Both the ERG b-wave and PERG P50-N95 amplitudes were significantly increased 20 min after blind spot stimulation, relative to 10 min post-stimulation and baseline. This effect was found selectively in myopic eyes, which showed significantly greater changes compared to non-myopic eyes. It was hypothesized that this finding might be associated with an increase in DAC activity via retrograde feedback from intrinsically photosensitive retinal ganglion cells (ipRGCs)^45^. It has previously been shown that melanopsin-containing axons of ipRGCs at the optic nerve head respond to stimulation with blue light matching the peak sensitivity of melanopsin^46^.

Melanopsin-expressing ipRGCs are a potential target system for physiological enhancement of dopamine levels in the myopic eye as they have been shown to project via their axon collaterals onto DACs^47^ and modulate the dopaminergic response to light^48,49^. The current study investigated how the electrophysiological response of the retina changes over longer periods of time after different protocols (i.e., durations) of short-wavelength blue light stimulation of the blind spot. It was hypothesized that longer stimulation protocols would lead to greater increases in the b-wave amplitude of myopic eyes and that this effect would be most pronounced 20 min after stimulation, as we have previously found^45^. We also tested the hypothesis that a linear relationship exists between axial length and the percent change in b-wave amplitude.

## Results

**Figure 1:**
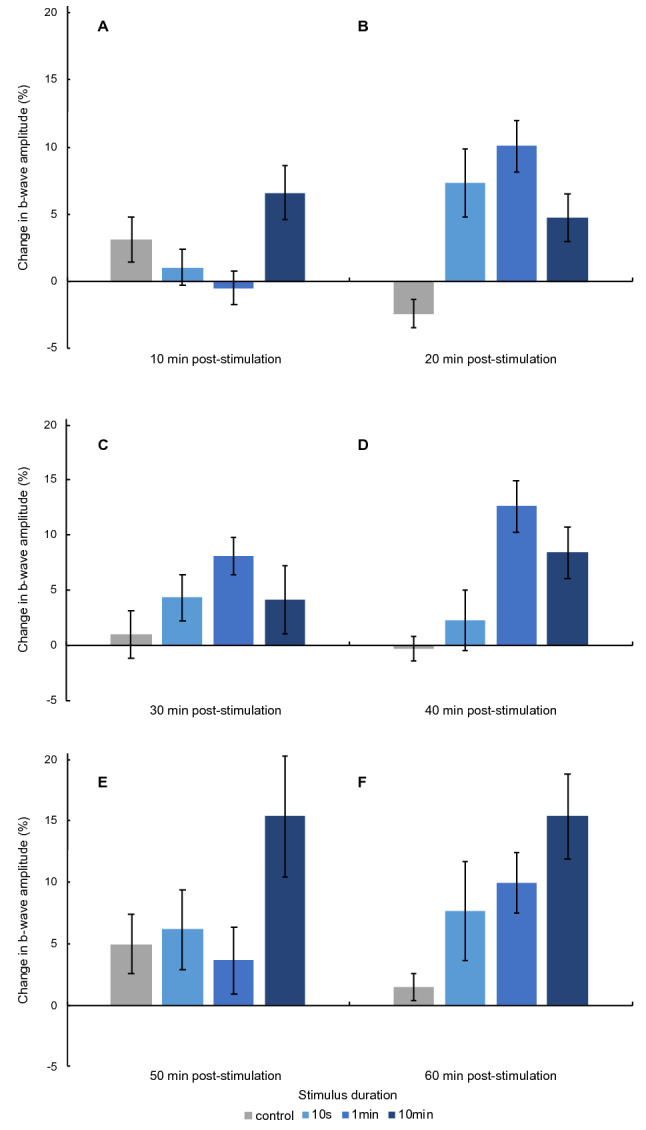
Mean and standard error of the mean (SEM) of the change in b-wave amplitude (%) relative to baseline after no stimulation and each stimulation protocol (10 s, 1 min, 10 min) are plotted at 10 min (**A**), 20 min (**B**), 30 min (**C**), 40 min (**D**), 50 min (**E**), and 60 min (**F**) after stimulation.

**Figure 2:**
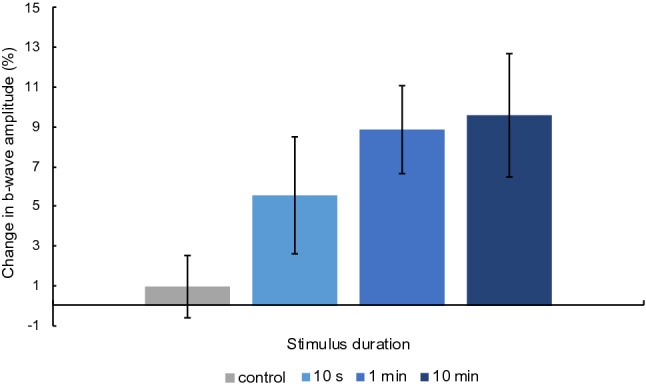
Mean and standard error of the mean (SEM) of the change in b-wave amplitude (%) relative to baseline after no stimulation and each stimulation protocol (10 s, 1 min, 10 min) averaged across measurements taken at 20, 30, 40, 50, and 60 min after stimulation.

In order to assess the overall impact of all blue light stimulation protocols relative to the control condition, the b-wave amplitude was normalized to baseline, and the percent change in amplitude was analyzed (Fig. 1). A 4 × 6 Bayesian repeated measures ANOVA with the factors “stimulation duration” and “time” revealed that the model including a main effect of stimulation duration was more likely than the null model (BF_10_ = 4.32), but this was not the case for the models including time (BF_10_ = 0.07) or both time and stimulation duration (BF_10_ = 0.33). Uncorrected post-hoc comparisons for the factor stimulation duration, collapsed across time, revealed a difference between all blind spot stimulation protocols and the control condition (Fig. 2) with weak evidence for 10 s (BF_10,U_ = 1.15), and positive evidence for 1 min (BF_10,U_ = 10.06) and 10 min (BF_10,U_ = 19.02) of stimulation. Collapsing across the four stimulation protocols the change in b-wave amplitude was compared at each measurement time. These post-hoc comparisons revealed weak evidence that the change in amplitude was elevated at 60 min compared to 30 min after blind spot stimulation (BF_10,U_ = 1.19).

### 10 s stimulation protocol

**Figure 3:**
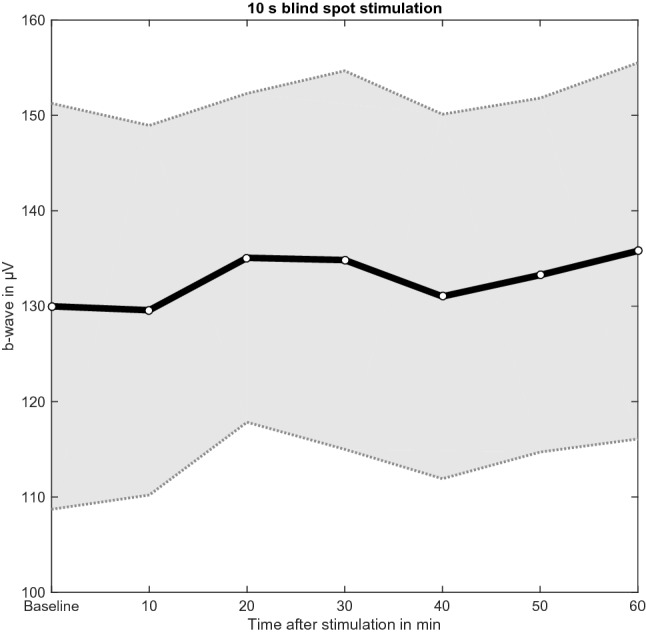
Mean and standard error of the mean (SEM) of the b-wave amplitude (µV) at baseline and 10, 20, 30, 40, 50, and 60 min after the 10 s blue light stimulation protocol.

Analysis of the alternative hypothesis for the 10 s stimulation condition revealed no evidence that the b-wave amplitude was significantly increased 20 min after stimulation for 10 s compared to baseline (BF_–0_ = 0.89). A repeated measures ANOVA revealed that the model that includes the main effect time was less likely compared to the null model (BF_10_ = 0.18). Uncorrected post-hoc comparisons found no evidence that the b-wave amplitude was larger 20 min after stimulation compared to 10 min post-stimulation (BF_10,U_ = 0.98, Fig. 3).

### 1 min stimulation protocol

**Figure 4:**
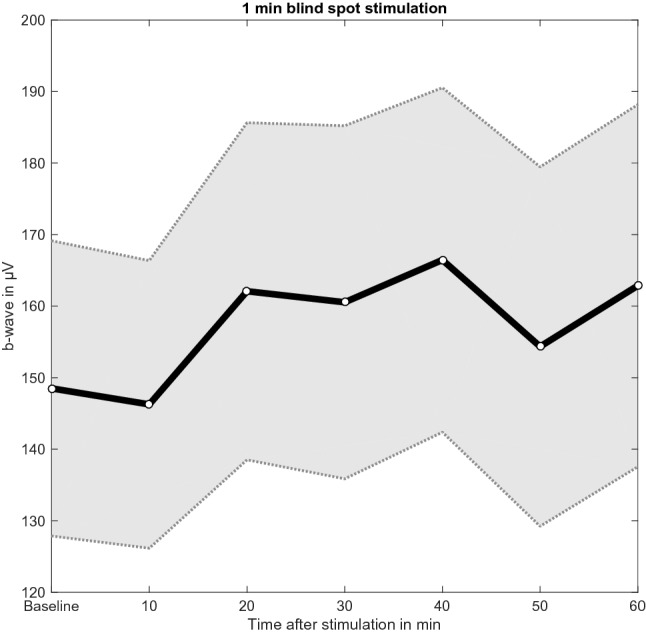
Mean and standard error of the mean (SEM) of the b-wave amplitude (µV) at baseline and 10, 20, 30, 40, 50, and 60 min after the 1 min blue light stimulation protocol.

Bayesian analysis confirmed the hypothesis that 20 min after 1 min of blind spot stimulation the ERG b-wave amplitude was increased compared to baseline, with weak evidence (BF_–0_ = 2.95). To explore the time course of the effect, a repeated measures ANOVA was conducted, which found positive evidence that the model including the main effect time was more likely than the null model (BF_10_ = 12.16). Uncorrected post-hoc comparisons showed weak evidence that at 20, 30, 40, and 60 min after stimulation the b-wave was significantly higher than baseline (BF_10,U_ > 1). This was not the case for the 10-min (BF_10,U_ = 0.43) and 50-min (BF_10,U_ = 0.46) post-stimulation measurements (Fig. 4). Further comparisons revealed positive evidence that the b-wave amplitudes at 20 min and 40 min post-stimulation were greater compared to 10 min after stimulation (BF_10,U_ > 3). There was also weak evidence that measurements 30 min and 60 min after stimulation were greater than at 10 min post-stimulation (BF_10,U_ > 1). Later in the time course weak evidence was found that the amplitudes at 20 min and 40 min post-stimulation were higher than 50 min after stimulation (BF_10,U_ > 1), and positive evidence that the b-wave amplitude at 60 min was higher than at 50 min post-stimulation (BF_10,U_ = 4.48).

### 10 min stimulation protocol

**Figure 5:**
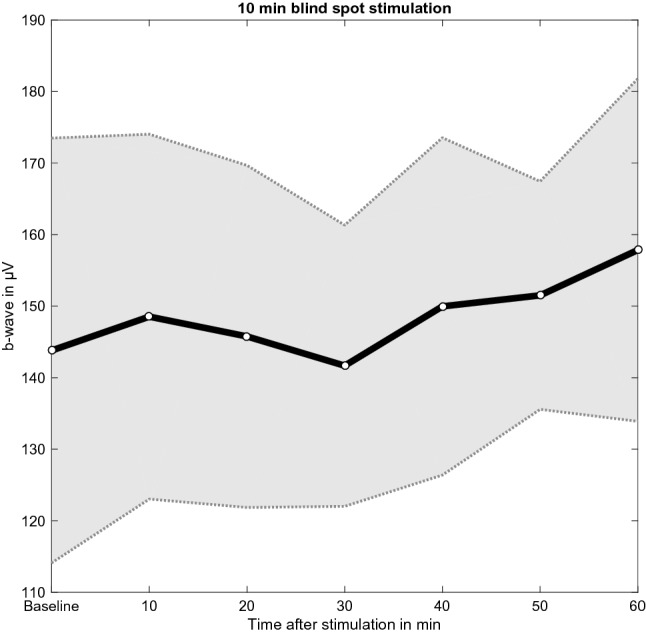
Mean and standard error of the mean (SEM) of the b-wave amplitude (µV) at baseline and 10, 20, 30, 40, 50, and 60 min after the 10 min blue light stimulation protocol.

After 10 min of blind spot stimulation no evidence was found that at the 20-min post-stimulation measurement the b-wave was elevated compared to baseline (BF_–0_ = 0.46). A Bayesian repeated measures ANOVA revealed that the model that includes the main effect time was less likely compared to the null model (BF_10_ = 0.29). Uncorrected post-hoc comparisons provided weak evidence that the b-wave amplitude was increased 60 min after blind spot stimulation, relative to baseline (BF_10,U_ = 1.08), 10 min (BF_10,U_ = 2.03), 30 min (BF_10,U_ = 2.67), and 40 min (BF_10,U_ = 2.11) post-stimulation, as well as positive evidence compared to 20 min (BF_10,U_ = 3.61) after blind spot stimulation (Fig. 5). There was weak evidence to support that the b-wave amplitude 50 min after stimulation was higher than 30 min (BF_10,U_ = 1.16) post-stimulation, and that measures at 40 min were higher than at 20 min (BF_10,U_ = 1.00) and 30 min (BF_10,U_ = 1.05) after blind spot stimulation.

### Control condition

**Figure 6:**
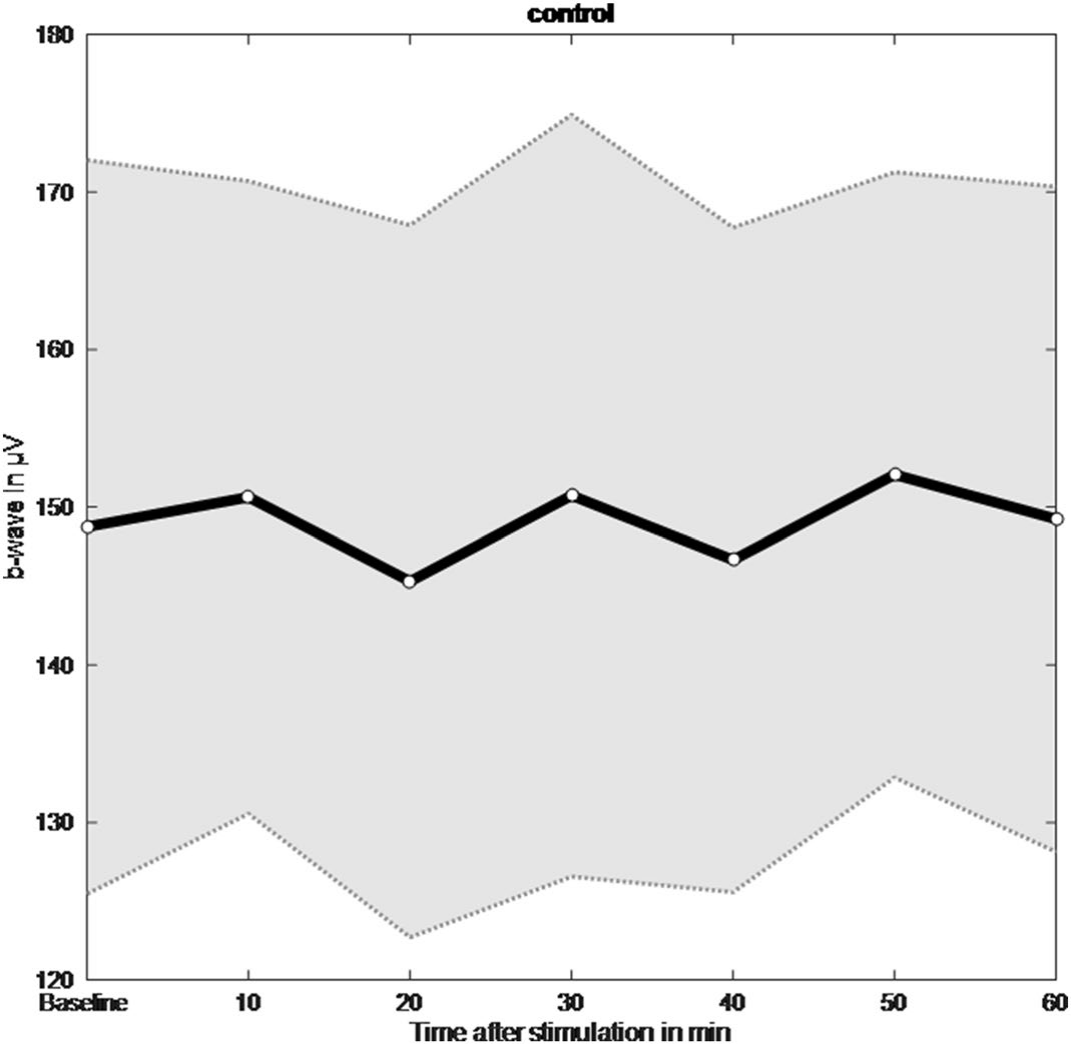
Mean and standard error of the mean (SEM) of the b-wave amplitude (µV) at baseline and 10, 20, 30, 40, 50, and 60 min after the no stimulation control condition.

Positive evidence was found for the null hypothesis that there was no change in the b-wave amplitude between baseline and the 20-min measurement (BF_0–_ = 4.59). A repeated measures ANOVA revealed positive evidence in favour of the model including a main effect of time compared to the null model (BF_01_ = 6.18). Corresponding post-hoc comparisons revealed weak evidence for the comparisons between b-wave amplitude at baseline and all time points measured after the control intervention (BF_01,U_ > 1; Fig. 6).

### Pupil

The mean pupil diameter for both eyes was calculated for each condition at baseline (control = 4.9 ± 0.5 mm; 10 s = 4.9 ± 0.4 mm; 1 min = 5.1 ± 0.4 mm; 10 min = 4.9 ± 0.4 mm) and after the last ERG measurement (control = 4.8 ± 0.4 mm; 10 s = 4.8 ± 0.4 mm; 1 min = 5.1 ± 0.4 mm; 10 min = 4.8 ± 0.3 mm). Paired *t*-tests revealed no significant differences between pupil diameter at baseline and after the control condition (*p* = 0.74) and each of the stimulation protocols (10 s: *p* = 0.23; 1 min: *p* = 0.78; 10 min: *p* = 0.30).

### Axial length and the b-wave

**Figure 7:**
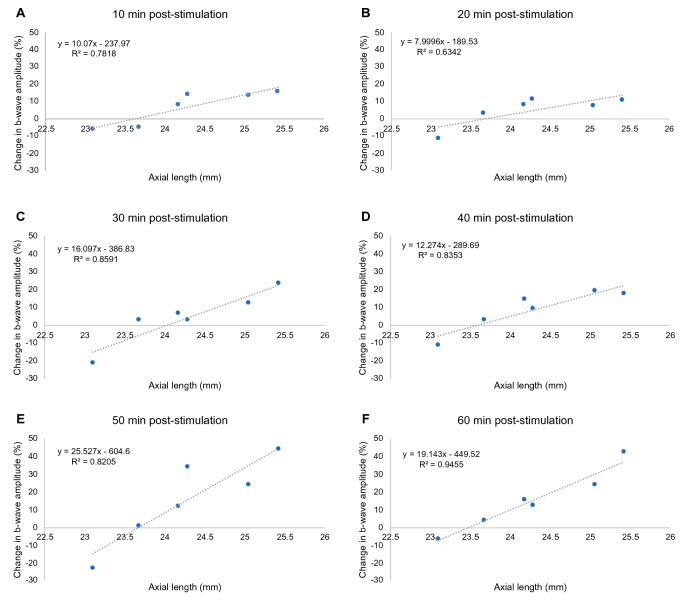
The relationship between axial length (mm) and change in b-wave amplitude (%) relative to baseline are plotted with a trend line for 10 min (**A**), 20 min (**B**), 30 min (**C**), 40 min (**D**), 50 min (**E**), and 60 min (**F**) after the 10 min stimulation protocol.

Bayesian linear regression analysis found weak evidence that absolute b-wave values were negatively associated with axial length at all measurement times for all stimulation protocols (BF_10_ > 1). Further investigation revealed that a positive linear relationship between axial length and change in b-wave amplitude was more likely than the null model with weak to strong evidence for all post-stimulation measurements (10 min: BF_10_ = 3.43, R^2^ = 0.78; 20 min: BF_10_ = 1.84, R^2^ = 0.63; 30 min: BF_10_ = 5.92, R^2^ = 0.86; 40 min: BF_10_ = 4.86, R^2^ = 0.84; 50 min: BF_10_ = 4.36, R^2^ = 0.82; 60 min: BF_10_ = 20.9, R^2^ = 0.95) for the 10 min stimulation protocol only (Fig. 7). Evidence for such an association was not found for the control, 10 s, or 1 min stimulation protocols. Inspection of the slope values of the linear association between axial length and percent change in b-wave amplitude for the 10 min protocol revealed a tendency toward larger slope values for longer post-stimulation times (10 min: slope = 0.10; 20 min: slope = 0.08; 30 min: slope = 0.16; 40 min: slope = 0.12; 50 min: slope = 0.26; 60 min: slope = 0.19).

## Discussion and conclusions

This study investigated the impact of varying the duration of blue light stimulation of the blind spot on the electrophysiological response of the myopic retina. All blue light stimulation protocols increased the b-wave amplitude relative to the no stimulation (control) condition and this increase was greater with longer stimulation time. Two distinct response patterns were observed for the 1 min and 10 min stimulation protocols. Specifically, a smaller but more rapid change in b-wave amplitude was measured after 1 min of stimulation. On the other hand, a larger but delayed increase in the b-wave amplitude was observed after 10 min of stimulation. These response patterns are considered in more detail below. Additionally, this response appears to be mediated by axial elongation, as a positive association was found between axial length and the percent change in b-wave amplitude for the 10 min stimulation protocol only. Together, these findings suggest that complex retinal mechanisms are engaged following short and long periods of stimulation of the blind spot with blue light.

Previous reasoning^45^ assumed that blue light stimulation of the blind spot activates the melanopsin-containing axons of ipRGCs located there^50^, which then retrogradely communicate with DACs^47^ to stimulate dopamine release. However, it cannot be excluded that the rods were stimulated indirectly by light scattered outside of the blind spot during stimulation. It has been shown that light-induced rod activity can mediate dopamine release in mice^51^. Of the many known functions of dopamine in the retina^52^, elevated retinal dopamine release is likely to impact horizontal and ON-bipolar cells. Ongoing dopaminergic activity could promote signalling throughout the ON-pathway. Given the importance of ON-bipolar cell activity to the b-wave response^32^, this may be the basis for the increase in b-wave amplitude observed following blue light stimulation. Hence, the increase in b-wave amplitude after blue light stimulation may reflect a dopamine-driven increase in ON-pathway activity. This is notable as there is accumulating evidence that ON-pathway activity may have a protective effect against myopia^29,30,53^.

Investigation of the time course of the effect of blue light stimulation on the b-wave amplitude revealed two distinct patterns: a rapid response and a delayed response. Compared to 10 min of stimulation, 1 min of blue light stimulation of the blind spot appears to have a smaller but more immediate effect on the ERG response. The observed peak in b-wave amplitude in the 1 min stimulation protocol is consistent with previous findings that similarly did not find a change in ERG or PERG amplitude until 20 min after blind spot stimulation^45^. The overall response pattern of the b-wave amplitude in the 60 min following 1 min of blind spot stimulation broadly resembles the change in the dopamine to 3,4-dihydroxyphenylacetic acid (DA:DOPAC) ratio of mice exposed to light. The DA:DOPAC ratio is an indicator of the rate of dopamine release. It has been reported that the DA:DOPAC ratio peaks after 1 min of light exposure, before decreasing at 5 min, and then returning to its peak and plateauing after 15 min of light exposure^51^. While a direct comparison between these two measures should not be made, it is nevertheless interesting that the change in the rate of dopamine release in response to continuous light shows similarities to the response pattern of retinal electrical activity following 1 min of blue light stimulation of the blind spot. The fact that the 1 min stimulation protocol showed this response pattern, but the 10 min protocol did not, could be a consequence of the amount of dopamine released. In future experiments, the light on blind spot induced dopamine release could be measured directly in addition to ERG recordings with further methods to prove this hypothetical connection.

Unlike 1 min of stimulation, 10 min of blue light stimulation of the blind spot does not result in a significant increase in b-wave amplitude until 60 min post-stimulation. The difference in response patterns after 1 min and 10 min of stimulation suggests that these stimulation protocols engaged two separate retinal mechanisms. Cell recordings have revealed that some ipRGCs can continuously respond to light for extended periods of time^54^. As a result, 10 min of stimulation likely leads to considerably greater dopamine release than 1 min of blue light stimulation of the blind spot. This is supported by the fact that the Bayes factor for the 10 min stimulation protocol is much larger than that for the 1 min protocol when each is compared to the control condition. An excess of dopamine released in response to 10 min of blue light stimulation of the blind spot could have contributed to the delayed change in b-wave amplitude.

Accumulation of extracellular dopamine can have an inhibitory effect on tyrosine hydroxylase, the rate limiting enzyme of dopamine synthesis, by acting on D2 autoreceptors located on DACs^55^. Therefore, the delay in b-wave amplitude change could reflect the time needed to clear the excess extracellular dopamine and release its inhibitory effect on tyrosine hydroxylase. Indeed, in the central nervous system, extracellular dopamine has been found to persist for prolonged periods of time^56,57^. However, further research is required to better understand the signalling cascades and cellular interactions that take place following blue light stimulation of the blind spot.

Lastly, correlational analysis revealed a positive association between axial length and the percent change in b-wave amplitude for the 10 min stimulation protocol only. This suggests that individuals with a longer axial length respond more strongly to the blue light stimulation. Importantly, this association was not seen for the absolute b-wave amplitude, which was negatively associated with axial length, in line with previous research^39,40^. Therefore, this finding is taken as a direct reflection of the effect of blue light blind spot stimulation on the b-wave response. This variation in the degree of the b-wave response within myopes may reflect upregulation of dopamine receptors with increasing axial length. When the availability or activity of a neurotransmitter is decreased, one way the system can respond is by upregulating the number of its receptors^58^. While a link between retinal dopamine and myopia has yet to be demonstrated directly in humans, animal models have associated experimental myopia with both decreased retinal dopamine levels^26^ and reduced DAC density^59^. Indeed, it has been reported that dopamine receptors are up- or down-regulated accordingly in response to changes in retinal dopamine levels^60^. However, further research is required to confirm these findings.

Alternatively, it could be that dopamine receptors in the myopic retina have an increased affinity to dopamine^61^. This is supported by the fact that apomorphine slows ocular growth and myopia development in form-deprived guinea pigs, which have significantly reduced retinal dopamine levels, but not control guinea pigs, which have normal dopamine levels^62^. Mutti et al.^63^ recently reported that repeated exposure to blue light (λ = 448 nm) resulted in greater, more sustained pupillary constriction in young non-myopic adults than in myopic adults. They proposed that this adaptation in the pupillary response could be due to increased melanopsin-mediated input in more hyperopic/less myopic eyes and that this could involve the retinal dopamine system^63^. Our results suggest that the potential to upregulate the dopaminergic electrical activity of the retina via melanopsin-containing ipRGCs might be greater in longer myopic eyes presenting chronically lower activity.

However, our study was limited in that there was no non-myopic control group and no long-wavelength light condition to control the short-wavelength blue light stimulus. Even though we performed a 10 min light-adaptation before each measurement, the increased b-wave due to over scattered light effects cannot be completely excluded. Since the participants were healthy, we assume that scattered light did not play a major role, although scattered light was not quantified. The control stimuli of a far-red light applied to the blind spot would function as a more definitive control than no stimuli. Despite the far-red light stimulation condition, which was not measured in the present study, a previous experiment using the same device and stimulation parameters as in the present study showed significant increase of choroidal thickness after brief stimulation of the optic disc with short-wavelength blue light compared to red-light stimuli or no-light stimuli condition (Hoseini-Yazdi et al.^64^). Future research should confirm the results of this study in a larger group of participants in order to verify their clinical significance and facilitate translation of the findings to the broader myopic population. The accumulating evidence^45^ that selective stimulation of the blind spot with blue light can increase retinal dopamine activity raises questions about the potential for such an intervention to slow myopia progression. The current results, which indicate that a treatment session as short as one minute could effectively stimulate the retinal dopaminergic system, further add to the clinical relevance of these findings. As such, a clinical trial may be an appropriate setting in which to confirm the results of this study.

Yet one should be careful when extending these findings to children. The current study investigated the retinal response to blind spot stimulation in young adults, although the target population for myopia intervention is typically children between the ages of six and 12 years^19,65^. There remains a need to better understand the functioning of the retinal dopamine system in children and its role in myopia onset and progression^14^. Unlike adults, there is no clear association between axial length and b-wave amplitude in children. Thus, different mechanisms may be involved at different stages of myopia progression. While, overall, it appears that there are some changes in retinal function in myopic children^66,67^, further research is required to determine whether they will respond in the same manner as young adults. In addition, our sample consists mostly of young women and we have not ruled out potential influences by hormonal changes during the menstrual cycle that have been previously documented^68^. In fact, we have not ruled out this factor, but considering that we used a photopic paradigm of stimulation, we do not anticipate that such potential changes might affect our results.

A potential limitation of the present study is the possible off-target blind spot stimulation due to loss of fixation. However, the stimulus size used was smaller than the average blind spot size to minimize the effect of microsaccadic movements. The blind spot stimulus and the fixation stimulus were shown on a black background, which is less attractive than the red fixation stimulus and helps to maintain fixation. Head movements are directly compensated for by the use of the virtual reality headset. Also, we used a special fixation cross called the ABC target (combination of bullseye and crosshair), which resulted in most stable fixation compared to other fixation targets in experiments that require stable fixation^69^ and participants were carefully instructed and advised to look at the fixation cross several times throughout the stimulation time. We acknowledge that off-target stimulation may be a more significant confounding factor with longer stimuli duration (i.e. 10 min). Still, the upregulation found after 10 min was also found at 1 min, and to a less extent after 10 s. Considering the aforementioned reasons, size of the stimulus spot, reinforcement of fixation by the examiner, and lack of reporting from participants about blue light being visible during treatment, eventual off-target stimulation should have a limited contribution, if any, to the effect found.

In conclusion, this study investigated the impact of different blind spot stimulation durations on the retinal electrical activity of myopic individuals. The results confirm the previous finding^45^ that stimulation of the blind spot with blue light increases activity in the inner plexiform layer of myopic individuals. The current study adds to this finding by reporting two different response patterns following 1 min and 10 min of blue light stimulation. Unlike 1 min of stimulation, which appears to engage a smaller, but more rapid response, 10 min of stimulation shows a larger, delayed peak in the b-wave amplitude. Notably, a positive linear association between axial length and percent change in b-wave amplitude was also found for the 10 min stimulation protocol. These findings may have implications for the treatment of myopia, as they indicate that even 1 min of selective stimulation of the blind spot with blue light can engage the retinal dopaminergic system, and that longer stimulation durations may act preferentially on longer eyes. Future research should continue to investigate the potential to upregulate retinal dopamine activity and slow myopia progression using targeted stimulation of the blind spot with blue light.

## Methods

This crossover exploratory study was conducted at the Clinical and Experimental Optometry Research Laboratory at the University of Minho (Braga, Portugal). This study follows similar methods to those previously reporting changes in the electrophysiological response of the myopic retina following stimulation of the blind spot with blue light^45^.

### Participants

Six healthy young myopic participants (4 female) without a history of ocular or systemic disease were recruited for the purposes of this study. Inclusion criteria were an age between 18 and 30 years, manifest refractions between − 0.75 diopters (D) and − 4.00 D, astigmatism below 1.00 D, and visual acuity of at least 0.00 logMAR with habitual correction. These participation criteria were used in order to minimize any influence of structural changes to the retina caused by axial elongation on the ERG response^70,71^. Mean age at participation was 24.3 years (SD = 4.7 years). Participants had a mean spherical refraction of − 1.63 ± 1.14 D, astigmatism of − 0.45 ± 0.49 D, and high contrast visual acuity (98%) of − 0.11 ± 0.05 logMAR and low contrast visual acuity (10%) of 0.02 ± 0.04 logMAR.

All participants provided written informed consent prior to their inclusion in this study, which was approved by the Ethics Committee for Health and Life Sciences at the University of Minho and conducted in accordance with the Declaration of Helsinki.

### Protocol

The complete experimental protocol consisted of virtual reality (VR) calibration of each individual’s blind spot position prior to ERG electrode placement, baseline full-field light adapted 3.0 ERG measurement, blind spot stimulation via smartphone Samsung Galaxy S7 screen (Samsung, Seoul, South Korea) with short-wavelength blue light (bell shaped spectrum with peak at λ = 450 nm, with a luminance of 14.74 ± 1.11 cd/m^2^)^45^, and post-stimulation full-field light adapted 3.0 ERG acquisitions. All measurements were taken between 10:00 am and 5:00 pm in order to avoid any influence of the circadian rhythm. Participants were asked to refrain from caffeine and nicotine consumption at least 5 h before each measurement. ERG acquisition, VR calibration, and blind spot stimulation were performed in accordance with the procedures of Amorim-de-Sousa et al.^45^ and ISCEV guidelines^72^ for full-field light-adapted ERG recordings. Briefly, before electrode placement, the skin was cleansed with an abrasive gel and then the gold-cup reference, the ground electrode and the active DTL-plus electrode (Dawson–Trick–Litzkow) were placed at 10 mm lateral to the outer canthus of the eye, at the central forehead and onto the lower fornix respectively. Impedance was checked before each measurement and recordings only taken when it was smaller than 5 kOhm. The full-field light-adapted 3.0 ERG test was then performed using the RETI-port/scan21 (Roland Consult, Wiesbaden, Germany) and consisted of a sequence of five single-flashes of white light (3.0 cd.s/m^2^) generated in a Ganzfeld stimulator against a light white background (30 cd/m^2^), with a stimulus rate of 0.625 Hz and a recording band pass filter of 0.3 Hz. As in previous studies^45^ to account for possible influences of mydriatic agents on the ERG response and to work under normal physiological conditions of pupil size, which is a critical factor to determine the treatment efficacy, measurements were recorded under undilated conditions. All the procedures took place under constant ambient lighting of approximately 400 lx measured with an illuminance meter (T-10A, Konica Minolta, Osaka) and pupil size was checked with an infrared pupilometer (VIP-200, NeurOptics, California) before each ERG measurements to ensure that luminance conditions were stable.

**Figure 8:**
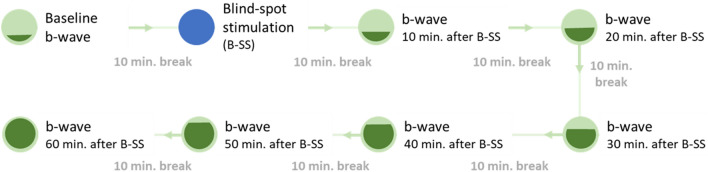
Timeline of measurements before (baseline) and after (10, 20, 30, 40, 50, and 60 min) each stimulation protocol. During each 10-min break participants underwent light-adaptation to Ganzfeld background illumination.

In order to evaluate the effect of different durations of blue light stimulation on the ERG response, four stimulation protocols were tested. These included a 1-min no stimulation control condition, as well as three blue light stimulation protocols: 10 s, 1 min, and 10 min. For the control condition, participants wore the VR headset and were asked to fixate on the same red cross as in the stimulation conditions but received no light stimulation. The red cross was an ABC fixation target, which is a combination of bullseye and crosshair with high fixation stability^69^. To assess the effect on the ERG response over time, recordings were taken at baseline and six times every 10 min following each of the four conditions (Fig. 8). Testing of the control condition and stimulation protocols took place in random order over the course of four visits and was always conducted within the same day period (i.e., morning or afternoon)—four individuals in the morning and two in the afternoon.

As it is unknown how topical mydriatics might influence the ipRGC response to blue light stimulation of the blind spot^73^, participants’ pupils were not dilated in this study. Therefore, in order to ensure that luminance conditions were stable, pupil size was measured using an infrared pupilometer (VIP-200, NeurOptics, California) at baseline and after the final ERG measurement for each condition.

### Statistical analysis

Data analyses were conducted on the b-wave amplitude measures of the light-adapted ERG cone response averaged across the two eyes of each participant. Paired *t*-tests were also performed on pupil size measurements taken before and after each condition.

The open source programs JAMOVI (1.1.9.0, jamovi project, 2019) and JASP (0.11.1.0, JASP Team, 2019) were used to conduct all statistical analyses. Bayesian inference statistics were used in order to test the hypothesis that an increase in b-wave amplitude would be observed 20 min after blind spot stimulation compared to baseline (hypothesis, BF_–0_). In the case of the control condition, analyses were performed to test the absence of an increase in b-wave amplitude relative to baseline (null hypothesis, BF_0–_).

Additionally, a Bayesian five-way repeated measures analysis of variance (ANOVA) was conducted for the control condition and each stimulation protocol (10 s, 1 min, 10 min). As previously an increase in b-wave amplitude was not observed until 20 min post-stimulation^45^, the timepoints after the 10 min measurement (i.e., 20, 30, 40, 50, and 60 min post-stimulation) were submitted to a 4 × 5 Bayesian repeated measures ANOVA with the factors “stimulation duration” and “time.” To investigate the relationship between axial length and b-wave amplitude, Bayesian linear analyses were conducted.

Interpretation of the Bayes factors was performed according to the degrees of evidence categories outlined by Kass and Raftery^74^. Namely, BF = 1–3 was interpreted as weak evidence, BF = 3–20 was considered positive evidence, BF = 20–150 indicated strong evidence, and BF > 150 was interpreted as very strong evidence.

### Ethical approval

This study was approved by the Ethics Committee for Health and Life Sciences at the University of Minho on 26/07/2019 (CEICV 038/2019) and conducted in accordance with the Declaration of Helsinki.

## Data Availability

The datasets generated during and/or analysed during the current study are available from the corresponding author on reasonable request.
